# Atypical spinal epidural lipomatosis: diagnostic strategy and treatment with AUSS technique—A case report

**DOI:** 10.3389/fsurg.2026.1980348

**Published:** 2026-09-29

**Authors:** Ke Deng, Leilei Wu, Long Peng, Quan Yuan, Changhua Peng, Xiao Yang, Chuan He, Xiaolin Li, Chaoju Zhang

**Affiliations:** Department of Orthopaedics, Jingzhou Hospital of Traditional Chinese Medicine, Jingzhou, China

**Keywords:** arthroscopic-assisted uniportal spinal surgery, atypical, diagnosis, spinal epidural lipomatosis, treatment

## Abstract

Spinal epidural lipomatosis (SEL) often presents with atypical symptoms, leading to misdiagnosis and delayed treatment, which significantly affects patients’ quality of life. The optimal treatment for SEL remains controversial. We report a case of SEL in a patient whose sole symptom was perianal pain—a presentation not previously documented in the literature. We summarize the clinical data and the use of arthroscopic-assisted uniportal spinal surgery (AUSS) for treatment, offering a novel approach for managing symptomatic SEL.

## 1 Introduction

SEL is characterized by abnormal accumulation of epidural adipose tissue within the spinal canal, resulting in compression of the spinal cord and nerve roots (1), Clinical manifestations depend on the involved segments and the degree of compression, commonly presenting as claudication, motor weakness, radiculopathy, or myelopathy (2), It is frequently misdiagnosed as lumbar disc herniation or spinal stenosis, and its optimal management remains under investigation.

We treated a patient with idiopathic lumbosacral SEL whose only symptom was perianal pain. The patient had visited multiple medical institutions for suspected anorectal diseases without a definitive diagnosis. After unsuccessful conservative treatment, the patient underwent AUSS-assisted laminectomy and spinal canal decompression. This case provides a new therapeutic option for symptomatic SEL.

## 2 Case presentation

A 71-year-old female presented with a 6-month history of perianal pain. She had consulted several hospitals for suspected anorectal conditions without relief. The pain progressively worsened, occurring after walking approximately 50 meters, characterized as a burning sensation, and resolving with rest. Physical examination revealed negative bilateral straight leg raise tests, with no motor or sensory deficits in the lower extremities. Past medical history was unremarkable, with no steroid use; body mass index (BMI) was 22.6 kg/m². The perianal Numeric Rating Scale (NRS) pain score was 7/10. Electromyography (EMG) showed moderate neurogenic injury in both lower limbs (radicular or higher-level involvement, predominantly affecting S1–S2), with chronic changes. Magnetic resonance imaging (MRI) revealed severe compression of the dural sac at L5/S1, exhibiting a “trefoil” sign (Figure 1).

**Figure 1:**
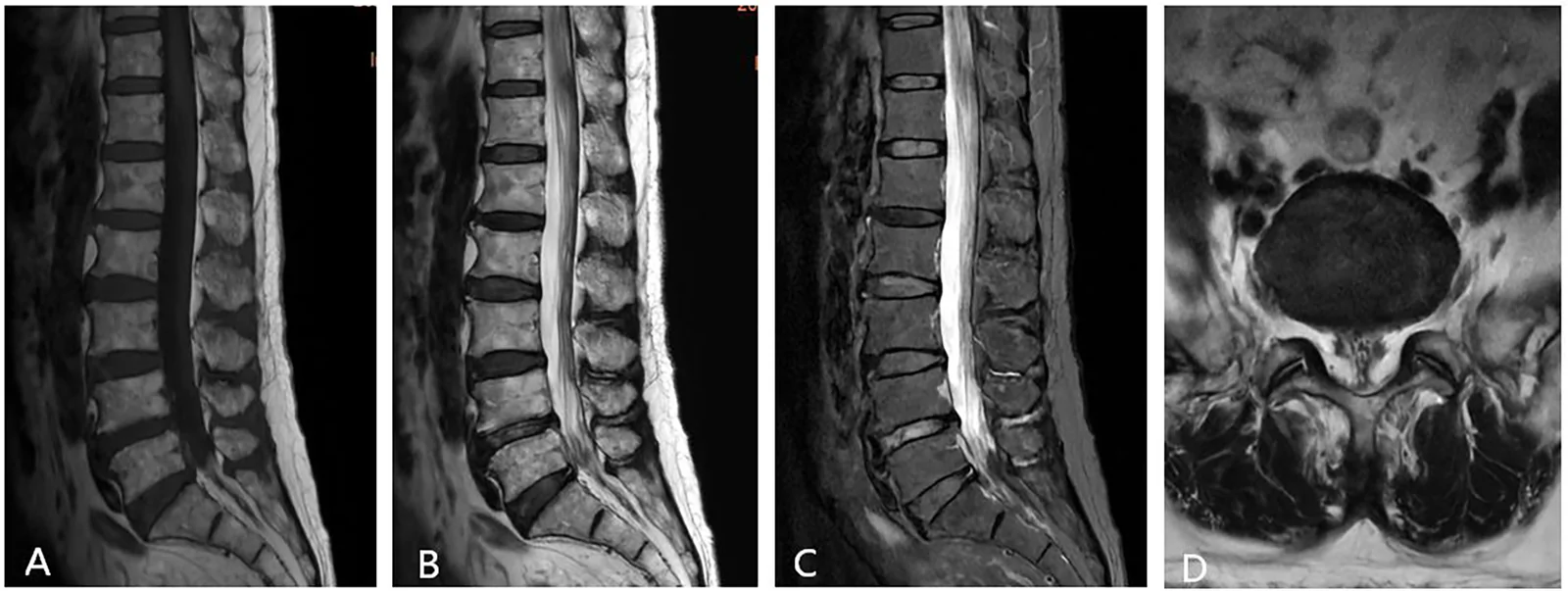
Preoperative lumbar MRI: **(A)** T1-weighted image; **(B)** T2-weighted image; **(C)** fat-suppressed image; **(D)** axial view at L5/S1.

Given the poor response to conservative treatment and the atypical symptoms, the patient underwent an L5/S1 epidural block (Figure 2). Post-procedural relief of perianal pain confirmed the symptomatic segment.

**Figure 2:**
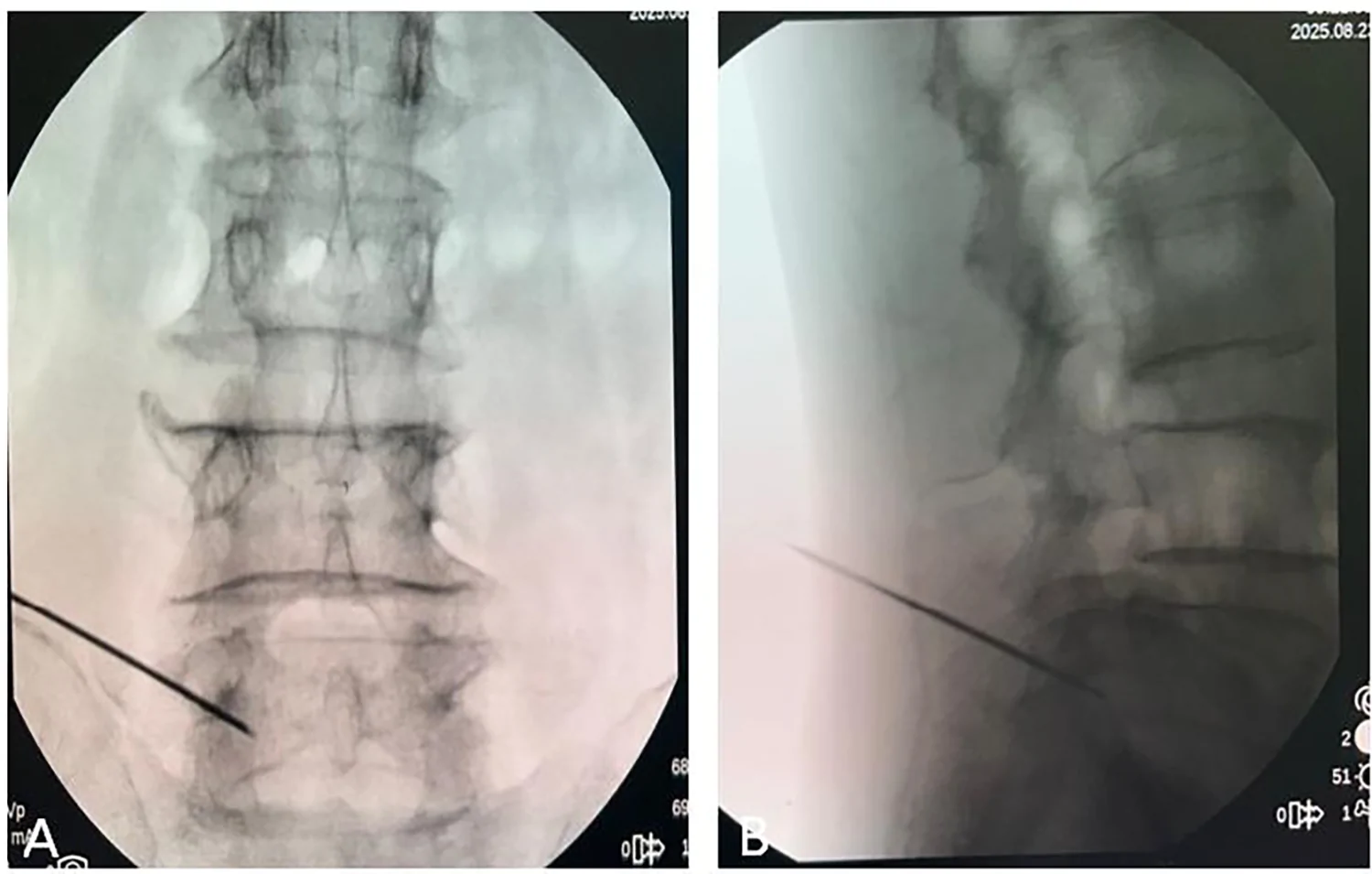
L5/S1 epidural block: **(A)** anteroposterior view, **(B)** lateral view.

## 3 Surgical technique

After general anesthesia, the patient was placed in the prone position with the abdomen free and the lumbar spine slightly flexed. The L5/S1 intervertebral space was identified via C-arm fluoroscopy and positioned perpendicular to the floor. The skin incision was marked at the intersection of the L5/S1 disc space and the medial border of the left L5/S1 pedicle line. The surgical field was then disinfected and draped. A 1.5 cm incision was made at the marked site, and dilators and separators were inserted to establish the working channel. An arthroscope and plasma radiofrequency probe were introduced (Figure 3). Under endoscopic visualization, the 90° plasma probe was used to remove soft tissue overlying the disc space. After adequate hemostasis and pre-hemostasis, the junction of the spinous process base and the L5 lamina, the medial edge of the facet joint, and the upper edge of the S1 lamina were exposed. A high-speed burr was used in a “circling” manner to thin the bony edges of the lamina, and Kerrison rongeurs were used to remove the medial lamina and facet margins, exposing the ligamentum flavum attachment. After radiofrequency hemostasis, the posterior ligamentum flavum was resected, and the contralateral bone was removed via an over-the-top approach. Intraoperatively, significant epidural fat hypertrophy was observed, with compression of the dural sac and bilateral S1 and S2 nerve roots. The epidural fat was resected to achieve decompression, fully exposing the dural sac and bilateral S1 and S2 nerve roots. Dural pulsations and relaxed nerve roots confirmed successful decompression (Figure 4). After thorough hemostasis, the wound was closed in layers (3, 4).

**Figure 3:**
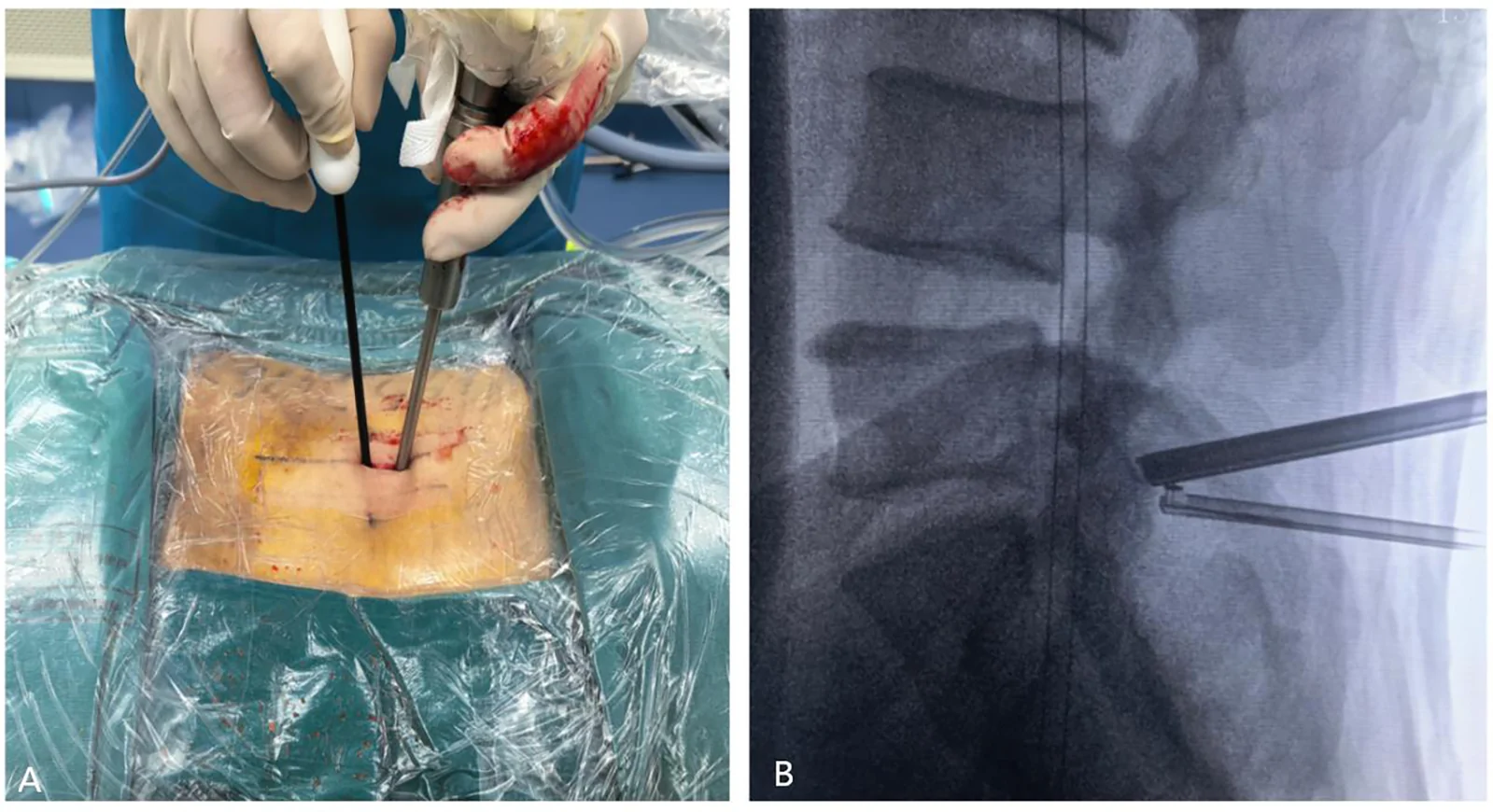
Intraoperative view of lumbar AUSS technique. (**A**) Intraoperative photograph showing the surgical procedure; (**B**) Intraoperative fluoroscopic view.

**Figure 4:**
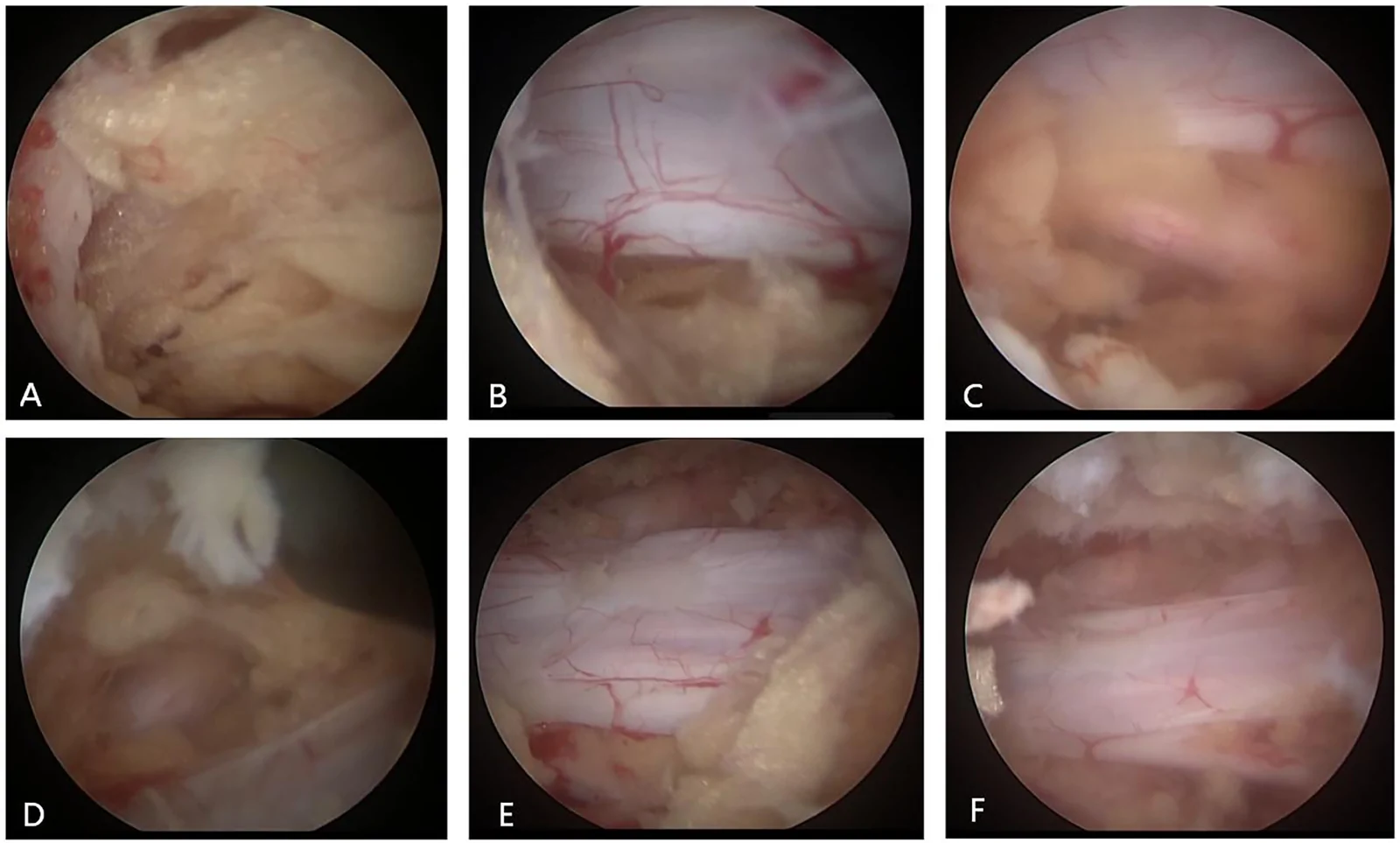
**(A)** Epidural fat hypertrophy; **(B)** Dural sac; **(C)** Left S1 nerve root decompression; **(D)** Right S1 nerve root decompression; **(E)** Post-decompression of bilateral S1 nerve roots; **(F)** Post-decompression of bilateral S1 and S2 nerve roots.

**Figure 5:**
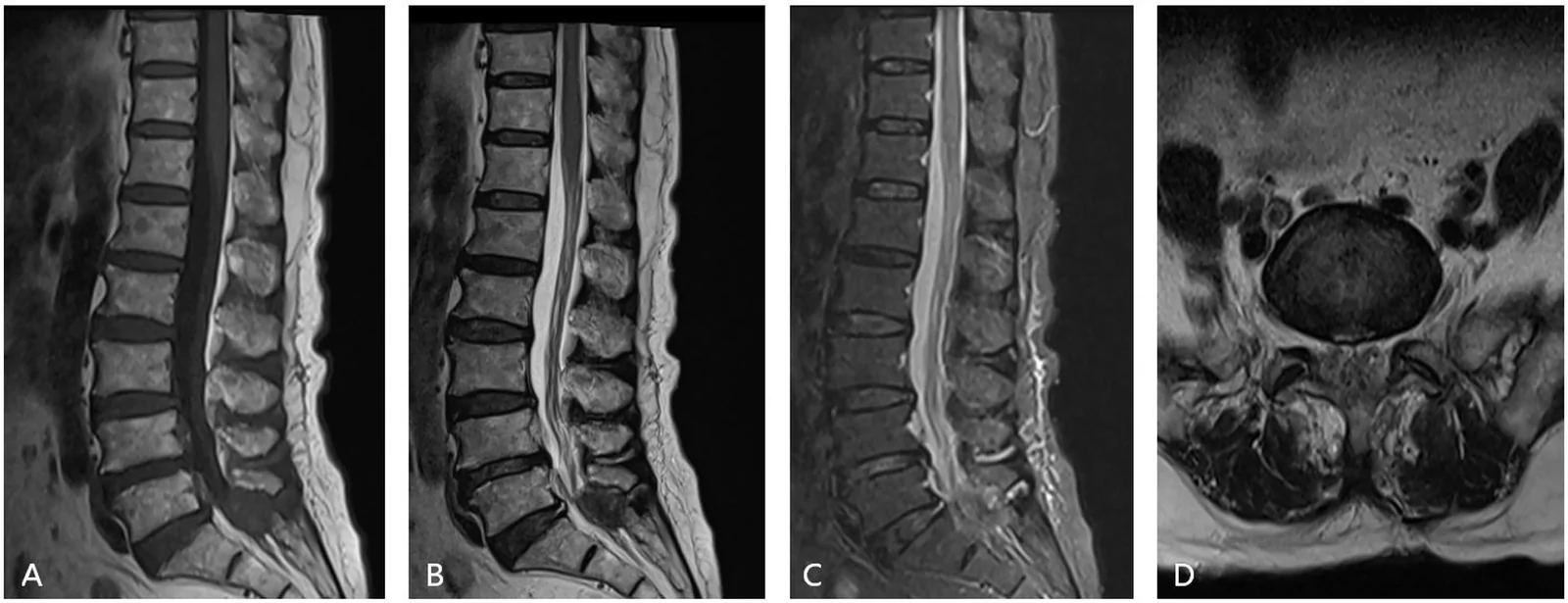
Lumbar MRI at 1 week post-operatively: **(A)** T1-weighted image; **(B)** T2-weighted image; **(C)** fat-suppressed image; **(D)** axial view at L5/S1.

One day postoperatively, the patient reported improvement in perianal pain compared with preoperative status. At discharge on postoperative day 7, perianal pain had significantly improved. Lumbar MRI obtained at 1-week postoperative follow-up demonstrated adequate decompression of the spinal canal at the L5/S1 level (Figure 5). At the 3-month telephone follow-up, the patient reported sustained pain relief and was satisfied with the treatment outcome.

## 4 Discussion

SEL has an extremely low incidence, and its pathogenesis remains unclear (5). It is currently believed to be associated with exogenous steroid use, obesity, endogenous steroid hormone disorders, prior spinal surgery, or idiopathic causes (6).

With the increasing use of steroids and rising global obesity rates, the prevalence of SEL is gradually increasing. However, symptomatic SEL patients may present with a variety of symptoms, depending on the location and degree of epidural fat compression. SEL often lacks specific symptoms, making it prone to underdiagnosis by radiologists and surgeons (7). MRI is considered the gold standard for evaluating SEL, as it can accurately differentiate signals from the dura mater and surrounding adipose tissue across different sequences, allowing precise measurement of fat thickness and extent (8).

Borrè et al (9) proposed four linear metrics based on axial lumbar MRI: the anteroposterior diameter of the dural sac (DuS), the anteroposterior diameter of epidural fat located ventrally and dorsally to the dural sac (EF), and the spinal canal diameter (SpiC). Based on the DuS/EF and EF/SpiC ratios, SEL is classified into four grades (Table 1). MRI facilitates early diagnosis of SEL, and based on the EF extent and clinical symptoms, appropriate treatment strategies can be selected.

**Table 1:** SEL grading criteria.

SEL grade	DuS/EF ratio	EF/SpiC ratio	Degree of growth	Clinical features
Grade 0 (normal)	≥1.5	≤40%	Normal	Asymptomatic
Grade I	1.0–1.49	41%–50%	Mild EF growth	Asymptomatic
Grade II	0.34–0.99	51%–74%	Moderate EF growth	Some cases symptomatic
Grade III	≤0.33	≥75%	Severe EF growth	All cases symptomatic

Currently, treatment for SEL is individualized. Options include conservative management (non-steroidal anti-inflammatory drugs, opioids, weight loss, steroid reduction, and activity modification) or surgical decompression when conservative measures fail, or when severe neurological deficits such as paraplegia, urinary retention, or significant neurological impairment occur (10). For obese patients, endocrine evaluation is recommended to rule out endogenous steroid disorders; bariatric surgery may help reduce epidural fat. For acute severe compression, early decompressive laminectomy and fat removal are indicated. With advances in endoscopic spinal surgery, several reports have described successful endoscopic decompression for symptomatic SEL (11).

In our case, perianal pain was the sole presenting symptom, with no evidence of peripheral neuropathy or anorectal pathology. Lumbar MRI revealed lumbar spinal stenosis with lumbosacral epidural lipomatosis and dural sac compression, presenting a trefoil or “Y” sign, consistent with the report by Kuhn et al (12). We measured a DuS/EF ratio of 0.69 and EF/SpiC ratio of 0.6 at the L5/S1 axial level, corresponding to Grade II SEL, which we considered symptomatic.

Given the unusual presentation of perianal pain as the sole symptom, we correlated the MRI and EMG findings and attributed the symptoms to compression of the bilateral S1 and S2 nerve roots by the hypertrophied epidural fat. The S1 and S2 nerve roots contribute to the pudendal nerve, with S2 being the dominant root supplying this nerve; lesions of S1 and S2 can thus cause perianal pain (13); Due to the atypical symptoms, epidural block were performed to confirm the diagnosis. After multiple failed conservative treatments, the patient strongly requested surgical intervention. Traditional laminectomy and fat resection require extensive paraspinal muscle dissection, leading to postoperative pain and delayed recovery. Endoscopic surgery, offering equivalent efficacy with less trauma, was preferred (14); As the patient had multilevel lumbosacral epidural lipomatosis requiring extensive fat resection and bilateral multi-root decompression, a broader visual field and decompression range were necessary. We ultimately chose the novel AUSS technique, which enabled bilateral S1 and S2 nerve decompression and epidural fat removal through a single 1.5-cm incision.

The AUSS technique was first described by Professor En Song in 2021, providing a novel approach for minimally invasive spine surgery. It combines the rotational flexibility of coaxial endoscopic spinal surgery with the triangulation operability of non-coaxial endoscopy, maximizing the surgical field and enhancing both the scope and precision of the procedure (15, 16). In our study, AUSS techniqueallowed extensive decompression through a single incision, with effective removal of lumbosacral epidural fat and bilateral S1/S2 nerve root decompression. Perianal pain improved postoperatively, with sustained symptom relief at final follow-up and no significant complications. Compared with unilateral biportal endoscopy (UBE), we found that AUSS technique offers several advantages for SEL requiring surgical intervention: (1) The single-incision design is not affected by patient obesity, reducing soft tissue dissection by 50% and minimizing soft tissue injury while preserving posterior column stability (3, 17);.(2) The unique non-coaxial rotation capability allows interchangeable use of the arthroscope and instruments in either hand, further expanding the decompression range and enabling multilevel decompression if needed; (3) AUSS technique better preserves the ligamentum flavum, reducing postoperative adhesions.

## 5 Conclusion

The optimal treatment for SEL remains controversial. We believe that AUSS technique offers a new therapeutic option for symptomatic SEL and provides valuable evidence for future development of optimal treatment strategies for SEL.

## Data Availability

The original contributions presented in the study are included in the article/Supplementary Material, further inquiries can be directed to the corresponding author/s.
